# Tamoxifen- and Triptorelin-Induced Major Hypertriglyceridemia: A Case Report

**DOI:** 10.7759/cureus.53779

**Published:** 2024-02-07

**Authors:** Widad Moussaoui, Fatima Zahra Lahmamssi, Hayat Aynaou, Houda Salhi, Hanan El Ouahabi

**Affiliations:** 1 Department of Endocrinology, Diabetology, Metabolic Diseases and Nutrition, Hassan II University Hospital, Fez, MAR

**Keywords:** breast cancer, fenofibrates, triptorelin, tamoxifen, hypertriglyceridemia

## Abstract

Tamoxifen, a selective estrogen receptor modulator (SERM), can have harmful side effects, such as hypertriglyceridemia, which can lead to acute pancreatitis. Meanwhile, triptorelin is an analog of natural GnRH (GnRHa), which may cause a small but significant increase in cholesterol and triglyceride (TG) levels.

We describe below the case of a patient with breast cancer treated with Patey’s operation, chemo-radiotherapy, and then with tamoxifen and triptorelin. After an exposure period of three months, she presented major hypertriglyceridemia at 56 g/L, total cholesterol at 13 g/L, LDL-cholesterol (LDL-C) at 4 g/L, and HDL at 0.25 g/L. The patient’s treatment was stopped by her oncologist. One month after starting an adapted diet and fenofibrate, her TG levels were reduced to 2 g/L.

We could confirm from these results that tamoxifen and triptorelin certainly modify lipid metabolism, hence the interest in evaluating the benefit-risk balance and regularly monitoring the lipid profile in order to avoid any fatal complication.

## Introduction

Hypertriglyceridemia is one of the most common lipid abnormalities that can cause acute pancreatitis. It usually occurs in patients with underlying disorders of lipoprotein metabolism and the presence of uncontrolled diabetes, alcohol misuse, or medication [1].

Tamoxifen and triptorelin are used in breast cancer therapy, with the disturbance of lipid balance as a possible side effect and bringing life-threatening complications, although it is rare.

We have encountered one case of asymptomatic major hypertriglyceridemia at 56 g/L in a patient with breast cancer, who was treated by Patey’s operation, chemo-radiotherapy, and then with tamoxifen and triptorelin. In this case report, we report the evolution of the lipid profile before and after the discontinuation of tamoxifen, the introduction of fenofibrate and an adapted diet, and the accountability of selective estrogen receptor modulators (SERMs) and triptorelin.

## Case presentation

We report the case of a 43-year-old female patient presenting with a history of breast cancer, having undergone Patey’s operation, chemotherapy, and radiotherapy. She was put on SERMs (tamoxifen: 20 mg/day) and an analog of natural GnRH (Decapeptyl, triptorelin: one injection/week). Her sister had undocumented hypertriglyceridemia on hygienic and dietary measures (Mediterranean-style diet, physical activity, and weight loss). However, a strong heredity of type 2 diabetes was noted.

One year earlier, before the start of adjuvant hormonal treatment, her treating oncologist noted a lipid disturbance (CT: 2.99 g/L; TG: 13 g/L; HDL: 0.25 g/L; LDL: 1.97 g/L) during a systematic check-up. The patient was prescribed dietary measures and then tamoxifen in May 2022.

The follow-up examination conducted three months after the start of the hormonal treatment showed a significant disorder of lipid metabolism with major hypertriglyceridemia at 56 g/L (Table 1). As a consequence, tamoxifen therapy was discontinued by her oncologist.

**Table 1:** Results of the different lipid tests performed on our patient TG, triglyceride; SERM, selective estrogen receptor modulator

	CT (g/L) (0.00-2.00)	TG (g/L) (<1.5)	HDL (g/L) (≥0.6)	LDL (g/L) (0.00-1.6)	CT/TG	TG/CT
Oct 25, 2021: before surgery	2.99	13	0.25	1.97	0.22	4.4 > 2.5; predominant hypertriglyceridemia
Aug 15, 2022: after one day of stopping SERMs	13	56	0.25	4	0.23	4.3 > 2.5
Aug 19, 2022: under fenofibrates	7.05	>47	0.28			
Aug 22, 2022	7.05	>18.39	0.31			
Aug 24, 2022	7.05	14.2	0.37			
Aug 26, 2022	7.05	14	0.35			
Sep 26, 2022	1.75	2	0.37	0.98		

Upon admission, the patient was in a healthy state and did not experience any abdominal or chest discomfort. The electrocardiogram (EKG) showed no irregularities, and the patient had a normal body weight with a body mass index (BMI) of 24.4 kg/m^2^. However, the patient did have an enlarged waist circumference (91 cm). The physical examination revealed the absence of goiter or acanthosis nigricans, as well as the absence of cutaneous, tendinous, or tuberous xanthomas on the elbows. There were no eruptive xanthomas observed on the buttocks, abdomen, and limbs, and no signs of xanthelasma or gerontoxon were present. Additionally, there were no indications of hypercortisolism.

**Figure 1:**
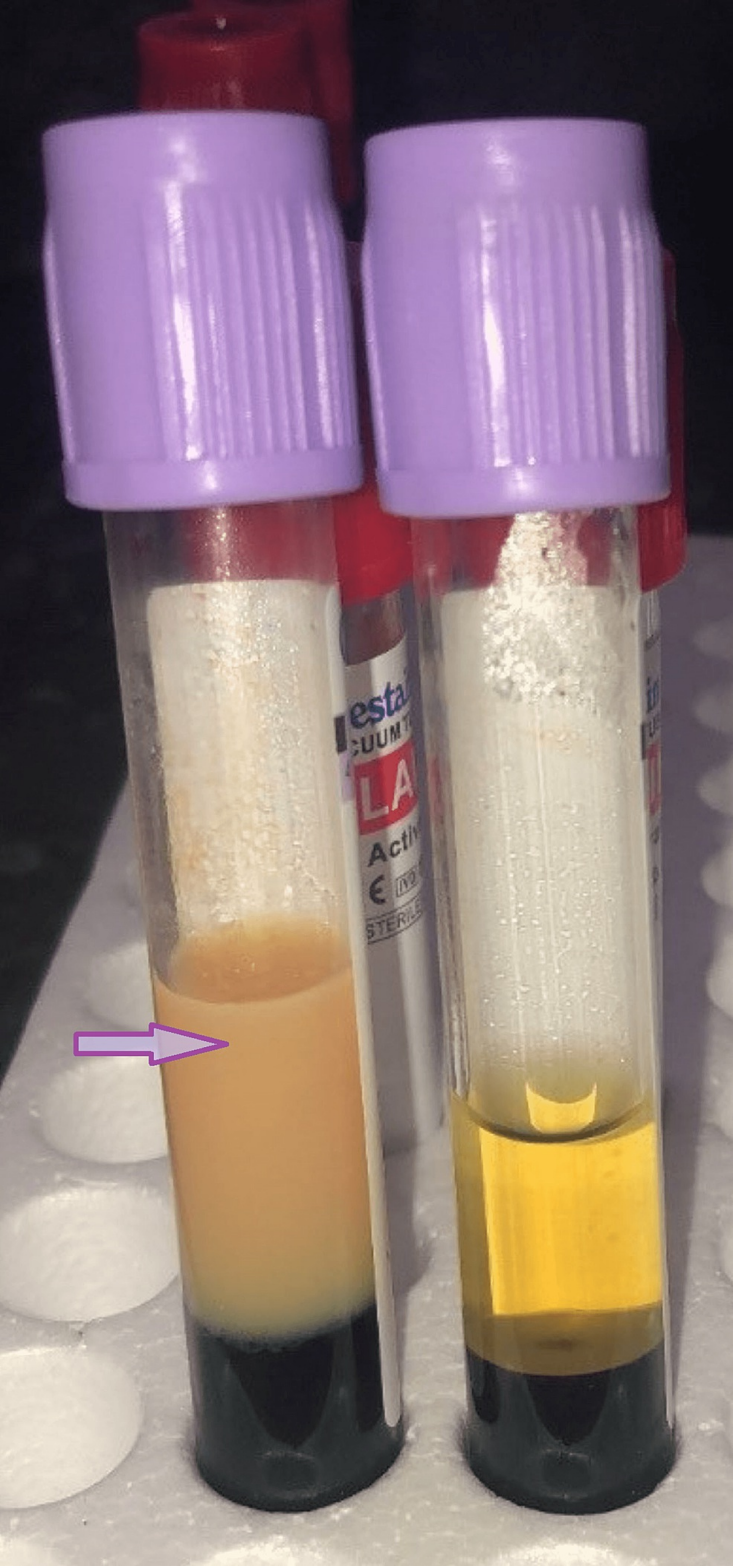
Aspect of our patient’s serum (purple arrow) in comparison with a normal serum

**Figure 2:**
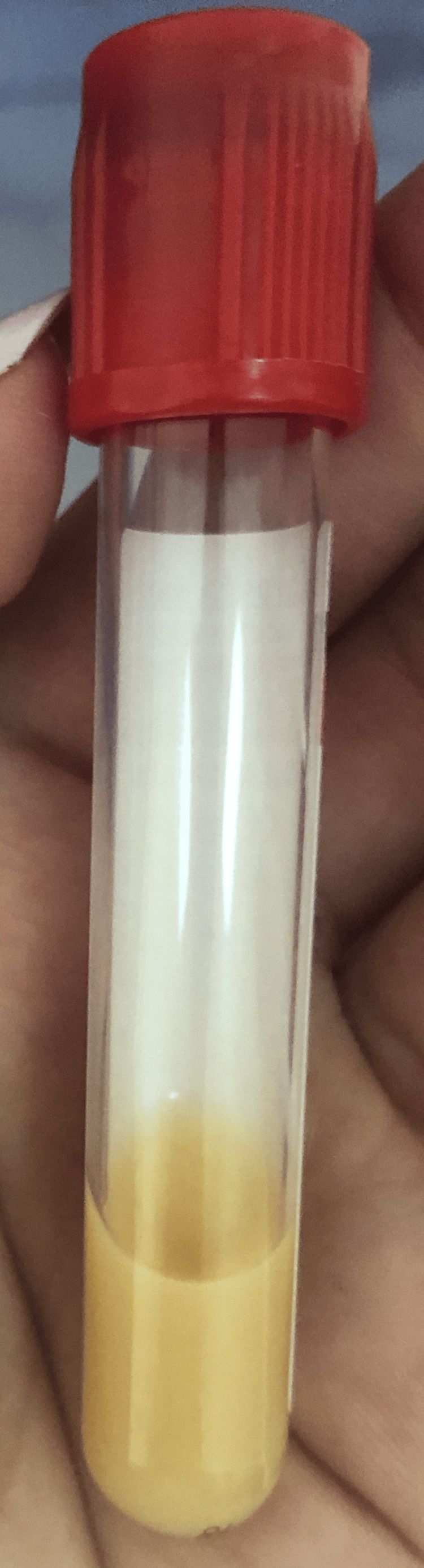
A negative 24-hour decantation test

We immediately conducted an emergency assessment, put the patient on a 72-hour liquid diet, and then on an adapted diet. In addition, we initiated treatment with fenofibrate (200 mg/day) with a progressive improvement of the lipid profile (Table 1). Lipasemia, hepatic, renal, and thyroid function tests and blood sugar levels were without abnormalities. Lipoprotein electrophoresis showed an absence of chylomicrons, alpha lipoproteins at 5.9% (15.1-39.9), pre-beta lipoproteins at 92.4% (2-31.2), and a negative 24-hour decantation test at 4° (Figures 1, 2). It is, therefore, probably a type IV hypertriglyceridemia.

After one month of treatment with fenofibrate 200 mg/day and well-conducted hygienic and dietary measures, as well as the discontinuation of tamoxifen, the lipid profile was checked again (Table 1).

The results of pharmacovigilance (Tables 2, 3) incriminate equally and with the same score of imputability I3B4 (French imputability method) for the two treatments: tamoxifen and triptorelin, which stands for an expected adverse drug reaction, and advised the stop of the two drugs in consultation with the prescribing doctor.

**Table 2:** Tamoxifen and triptorelin intrinsic imputability study R0, not performed or inconclusive; C2, plausible; L0, not available or inconclusive investigation; S2, plausible; I3, likely

	Tamoxifen	Triptorelin
Time of occurrence of the event	Compatible time frame	Compatible time frame
De-challenge	Suggestive evolution	Suggestive evolution
Re-challenge	R0	R0
Chronological Score (C0-C3)	C2	C2
Semiotics	Semiology not suggestive of a pharmacological role	Semiology not suggestive of a pharmacological role
Non drug causes (confounders)	Etiological assessment	Etiological assessment
Laboratory investigations	L0	L0
Semiological Score (S1-S3)	S2	S2
Intrinsic Score (I0-I6)	C2S2 = I3	C2S2 = I3

**Table 3:** Tamoxifen and triptorelin extrinsic imputability B, extrinsic score; B4, expected adverse drug reaction

	Score
Tamoxifen	Notable effect referenced in RCP and VIDAL: score B4
Triptorelin	Notable effect referenced in RCP and VIDAL: score B4

## Discussion

We faced the case of a 43-year-old woman under tamoxifen and triptorelin as a treatment for her breast cancer. She presented major hypertriglyceridemia at 56 g/L with no complications, such as acute pancreatitis, as a side effect after three months of therapy.

Tamoxifen, a SERM, is widely used for hormone therapy of estrogen receptor (ER)-positive breast cancer. It has tissue-specific agonist and antagonist properties [2]. It can disturb the lipid balance by increasing VLDL (very low-density lipoprotein) synthesis and inhibiting lipoprotein lipase (LPL) and hepatic triglyceride lipase (HTGL) synthesis. This disturbance is more pronounced in the presence of predisposing factors [3]. Previous reports have shown that estrogen impairs the metabolism and clearance of triglyceride (TG)-rich lipoproteins due to post-heparin lipolytic activity suppression [4,5]. Post-heparin lipolytic activity consists of two activities: HTGL and extrahepatic LPL. 

Triptorelin, a synthetic hormone, is an analog of natural GnRH (GnRHa). It can be used in early breast cancer in pre-menopausal women, endometriosis, and also in men with advanced prostate cancer. It has been associated with a small but significant increase in cholesterol and TG levels.

Similar to our patient’s case, individuals with familial hypertriglyceridemia or familial combined hyperlipidemia have been reported to have very high serum TG levels, which can lead to serious consequences [6,7]. Contrasting with a moderate rise in TGs in normolipidemic patients before the start of SERMs.

Tamoxifen is more likely to increase TG levels in patients with predisposing factors, such as an elevated pre-prescription TG levels, diabetes, obesity, chronic renal failure, non-alcoholic fatty liver disease, alcohol abuse, concomitant use of certain medications, familial hypertriglyceridemia, and combined hyperlipidemia [8].

Screening for dyslipidemia prior to tamoxifen administration is therefore strongly recommended, and predisposed patients should have regular lipid monitoring [9]. If abnormally high levels are found, tamoxifen should be discontinued in consultation with the prescribing doctor to avoid the risk of severe acute pancreatitis.

In the case we reported, we observed an acute increase of TG after tamoxifen use in a patient who already had hypertriglyceridemia. In Table 4, we report some cases of hypertriglyceridemia under tamoxifen found in the literature.

**Table 4:** A few cases of hypertriglyceridemia under tamoxifen reported in the literature

Author	Age (years)	History of dyslipidemia	Triglyceridemia (mg/dL)	Onset time (months)
Noguchi et al. [10]	34	Unspecified	3673	7
Colls and George [7]	44	Yes	6984	Unspecified
Elisaf et al. [11]	53	Yes	5200	8
Artac et al. [12]	51	Unspecified	1344	12
Lin et al. [13]	43	Yes	1040	24
Alagozlu et al. [14]	46	Yes	900	12
Sakhri et al. [15]	44	Yes	1180	12
Brun et al. [6]	61	Yes	2790	Unspecified
Isobe et al. [9]	47	Unspecified	1881	5
Hozumi et al. [16]	49	Unspecified	1572	11
Hozumi et al. [16]	54	Unspecified	1123	3
Hozumi et al. [16]	49	Unspecified	2402	12
Khabbal et al. [17]	44	No	1000	10

However, a small but significant increase in cholesterol and TG levels was observed in one study after GnRHa treatment in women with endometriosis [18]. All values were within normal limits (Table 5).

**Table 5:** Lipid profile before and after treatment with triptorelin n = number of women with endometriosis treated with triptorelin *p < 0.05; **p < 0.001; n = 43

Lipid	Before	After
Cholesterol (mmol/L)	4.82 ± 0.14	5.31 ± 0.15**
Triglyceride (mmol/L)	0.96 ± 0.06	1.11 ± 0.07*
HDL cholesterol (mmol/L)	1.35 ± 0.05	1.41 ± 0.05

We promptly put the patient on a 72-hour liquid diet and then on an adapted diet. After eliminating the contraindications of fibrates, we initiated treatment with fenofibrate (200 mg/day), resulting in a progressive improvement of the lipid balance. Fenofibrates increase lipolysis and the elimination of atherogenic TG-rich particles from plasma by activating the LPL and reducing the apoprotein CIII production.

In the literature, several management options for hypertriglyceridemia have been described, ranging from a liquid diet, a hypolipidemic, hypocaloric, and a high protein diet to drug treatment with fibrates. Gemfibrozil is the most hypotriglyceridemic, followed by bezafibrate, fenofibrate, and ciprofibrate [19].

The combination of fibrates and statins is sometimes necessary and reduces cardiovascular risk [20], and it is appropriate in severe mixed dyslipidemia. Only the combination of statins with gemfibrozil is not recommended because of the risk of rhabdomyolysis [21].

The hypotriglyceridemic effect of Omega 3 has been proven, but it only occurs at high doses (2-4 g/day) [22] and leads to a 30-50% decrease in TGs. The disadvantage of Omega 3 “drugs” is their relatively high cost. Consumption of fatty fish is an alternative, but at a dose of one box per day, digestive tolerance is sometimes poor.

Volanesorsen is a new molecule that can be indicated in major hypertriglyceridemia with a very high risk of acute pancreatitis [23]. Approach and compass studies have been shown to lower TGs and suppress the recurrence of acute pancreatitis [24,25].

Plasmapheresis is reserved for salvage situations in cases of refractory major hypertriglyceridemia associated with threatening hypertriglyceridemic acute pancreatitis [26].

## Conclusions

We could confirm from these results that tamoxifen and triptorelin certainly modify lipid metabolism, hence the interest in evaluating the benefit-risk balance before their administration and constantly monitoring the lipid profile during the treatment, in order to avoid any complications, such as acute pancreatitis, which is the most characteristic complication of major hypertriglyceridemia, myocardial infarction, or stroke.
